# Study of the Sensitivity of DC Arc Temperature Field, Pressure Field, and Potential to Process Parameters

**DOI:** 10.3390/mi16080919

**Published:** 2025-08-09

**Authors:** Yongjun Liu, Gaosong Li, Shuai Zhang, Zhenya Wang

**Affiliations:** 1 School of Intelligent Manufacturing, Huanghuai University, Zhumadian 463000, China; chengquan33@126.com (Y.L.); shuai89_zhang@126.com (S.Z.); 2 Department of Mechanical Engineering, Tsinghua University, Beijing 100084, China

**Keywords:** DC arc, numerical modelling, pressure field, temperature field, sensitivity

## Abstract

DC arcs are widely used in many fields such as shipbuilding, machinery manufacturing, and aerospace due to their advantages of high energy density, simple structure, and low price. However, there are few studies on the sensitivity of the arc pressure and temperature fields to current and protective gas flow rate. In order to solve this problem, this paper establishes a numerical model for the coupling of DC arc electric–thermal–flow multi-physical fields. Based on this model, the variation rules of the arc temperature, pressure, and potential field with current or protective gas flow rate are studied, respectively, when the current is 100–600 A or the gas flow rate is 18–48 L/min. The results show that the current is the most important factor in the sensitivity of the arc temperature and potential field to the current and protective gas flow rate. With the increase in current, the Joule heat power increases significantly, and the arc central temperature shows a nonlinear increase to 27,000 K. With increasing current, the peak of the pressure field gradually shifts to the region below the top of the wire arc, and the highest pressure increases by 14 times. When the current is small, the increase in argon flow rate can inhibit the spreading of the temperature field by forced convection; when the current is large, the arc contraction with an increasing argon flow rate leads to an anomalous increase in the arc-central temperature. In addition, the energy accumulation mechanism in the strong-current–high-flow-rate coupling region is also revealed, a coupled mathematical model of arc contraction and turbulent loss under the Lorentz force is constructed, and the thermodynamic properties of the arc under the coupling of multi-physical fields are elucidated.

## 1. Introduction

The DC arc, as a high-energy-density discharge phenomenon, is widely used in the fields of shipbuilding, machinery, and aerospace because of its advantages of simple operation, cheap equipment, and high welding quality [1,2,3]. In practical applications, the current and shielding gas flow rate have a great influence on the temperature and pressure fields of the arc, as well as the flow rate of the plasma [4,5]. However, most of the current studies on the electric arc speculate on the laws governing the influence of the arc process parameters on the temperature and pressure fields during the arc welding process by measuring the mechanical and microstructural dimensions of the weldments [6,7,8]. This inevitably introduces new errors due to the homogeneity of the measurers and materials as well as the variability of the experimental equipment, which has obvious limitations and uncertainties, while consuming a large amount of human and physical resources [9,10]. Therefore, a new method that can intuitively reflect the arc temperature field, pressure field, and plasma flow velocity should be explored. This will not only speed up the adjustment of process parameters and provide a more intuitive understanding of the mechanism of action of process parameters on the arc but also reduce the number of experiments [11].

Numerical modelling has received widespread attention as a new method that can serve the traditional experiments, which are unable to visualise the temperature and pressure fields of the arc. For example, Al-Nasser et al. established a compressible and incompressible numerical model of the electric arc and investigated the influence of compressibility on the behaviour of the arc [12], and Baeva et al. established a one-dimensional arc plasma prediction model and investigated the variation of the spatial distribution of the temperature of electrons and heavy particles with the gap [13]. In recent years, in numerical simulations, scholars have gradually coupled the temperature and fluid fields [14,15,16]. However, the systematic study of the mechanism of the multi-field coupling action in the process is not deep enough, especially the coupling between the electric field and plasma turbulence is not clear.

In order to solve this problem, many scholars have established numerical models of arc welding. Jiang et al. established a numerical model of a shielding gas nozzle and studied the effect of the number of nozzles on the temperature and pressure fields of the arc [17]. Li et al. established a magnetohydrodynamic model of a high-current vacuum arc and investigated the effect of a transverse magnetic field on the distribution of the plasma, with the result that the surface magnetic field could change the arc’s symmetry [18]. Wang et al. established a numerical model of a single-wire and double-wire arc and predicted the temperature, velocity, and pressure distribution characteristics of the arc [19]. Fan et al. established a high-current MIG welding model and investigated the effect of an external magnetic field on the temperature, with the result that the surface external magnetic field could compress the arc [20]. Wei et al. developed a numerical model of magnetohydrodynamics to investigate the effect of different gaps on the arcing problem and verified it experimentally [21]. Zhao et al. established a numerical model of a magnetic field-assisted arc; as a result, the surface arc can compress the arc form and reduce the temperature field of the arc [22]. Wang et al. established a numerical model of the arc and investigated the temperature field, pressure field, and velocity field within the argon arc, with results showing that a reasonable arrangement of the longitudinal magnetic field is conducive to improving the stiffness of the arc [23]. Tang et al. established a temperature field model of the arc material to study the temperature field of the arc discharge, and the results revealed significant differences in the arc temperature of different materials [24].

The above studies have made a great contribution to the development of multi-physical field coupling in electric arcs, although they have solved the shortcomings of traditional experiments, which cannot intuitively capture the distribution characteristics of temperature, pressure, and velocity fields in electric arcs. However, the current research on the sensitivity of temperature and pressure fields to current and protective gas flow rate is not deep enough. In order to solve this problem, this paper establishes a multi-physics field coupling analysis and numerical simulation platform and investigates the influence patterns of the current and protective gas flow rate on the temperature field, pressure field, and plasma flow velocity. At the same time, the sensitivity of the temperature field and pressure field to different levels of current and protective gas flow rate is also studied. Finally, the relative sensitivity of the arc to the current and protective gas is analyzed from both the temperature field and pressure field.

## 2. Numerical Model

### 2.1. Modelling and Assumptions

**Figure 1:**
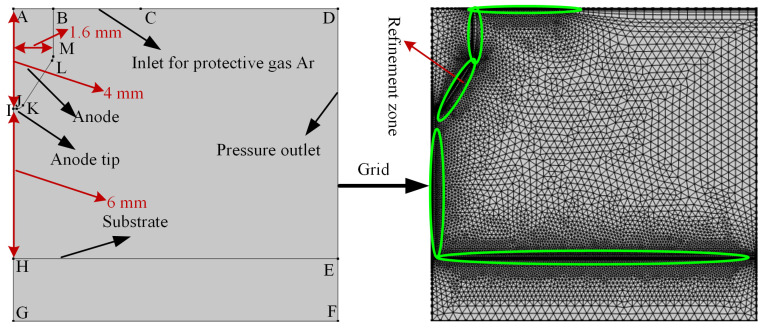
Numerical model and grid division of tungsten TIG welding arc.

The numerical model of tungsten TIG welding arc is shown in Figure 1. The inner diameter of the tungsten electrode is 1.6 mm; the length is 4 mm; the tip of the tungsten electrode is in the shape of a circular arc; and the distance from the welding plane is 6 mm; the maximum unit at the boundary is 0.0838 mm; the number of boundary layers is 6; the mesh type is triangular mesh; and the tungsten electrode shielding gas is high-purity argon gas.

The following assumptions are made to simplify calculations:

(1) The arc is in a steady state and is two-dimensional axisymmetric;
(2) The effect of the anode surface state on the arc is not considered;
(3) The arc space plasma is locally in thermal equilibrium;
(4) The arc iso-discrete flow state is laminar;
(5) The external environment is standard atmospheric pressure, argon each physical property is only a function of temperature;
(6) The tungsten electrode tip current density is uniformly distributed.

To study the mechanism of action of process parameters on the arc, the actual DC arc was simplified, which inevitably limits the applicability of the model. That is, based on the above assumptions, the model proposed in this paper is only applicable to steady-state conditions, low current, arc length less than 10 mm, and environments where the shielding gas is an inert gas (such as argon or nitrogen). It is not suitable for transient arcs, arcs with larger lengths, arc ignition and extinction, or arcs containing metal vapours.

### 2.2. Control Equations

Control equations for solving the two-dimensional axisymmetric arc model are presented. The set of control equations includes the mass, momentum and energy conservation equations, and the mass conservation equation is shown in Equation (1).

(1) $$\frac{\partial \rho }{\partial t}+\frac{1}{r}\frac{\partial \left(r\rho v\right)}{\partial r}+\frac{\partial \left(\rho u\right)}{\partial z}=0$$

where *r* and *z* are radial and axial coordinates, respectively; *u* and *v* are axial and radial velocities, respectively; and *ρ* is the density of argon gas.

The equation for the conservation of radial momentum is shown in Equation (2).

(2) $$\frac{\partial \left(\rho v\right)}{\partial t}+\frac{1}{r}\frac{\partial \left(r\rho v^{2}\right)}{\partial r}+\frac{\partial \left(\rho uv\right)}{\partial z}=F_{r}-\frac{\partial P}{\partial r}+\frac{1}{r}\frac{\partial }{\partial r}\left(2\mu r\frac{\partial u}{\partial r}\right)-2\mu \frac{v}{r^{2}}+\frac{\partial }{\partial z}\left(\mu \frac{\partial v}{\partial z}+\mu \frac{\partial u}{\partial r}\right)$$

where *P* is the gas pressure, and *μ* is the argon viscosity.

The equation of conservation of axial momentum is shown in Equation (3).

(3) $$\frac{\partial \left(\rho u\right)}{\partial t}+\frac{1}{r}\frac{\partial \left(r\rho uv\right)}{\partial r}+\frac{\partial \left(\rho u^{2}\right)}{\partial z}=F_{z}-\frac{\partial P}{\partial z}+\frac{1}{r}\frac{\partial }{\partial r}\left(\mu r\frac{\partial v}{\partial z}+\mu r\frac{\partial u}{\partial r}\right)+\frac{\partial }{\partial z}\left(2\mu \frac{\partial u}{\partial z}\right)$$

The equation for the conservation of energy is shown in Equation (4).

(4) $$\frac{\partial \left(\rho c_{p}T\right)}{\partial t}+\frac{1}{r}\frac{\partial \left(r\rho vc_{p}T\right)}{\partial r}+\frac{\partial \left(\rho uc_{p}T\right)}{\partial z}=\frac{1}{r}\frac{\partial }{\partial r}\left(kr\frac{\partial T}{\partial r}\right)+\frac{\partial }{\partial z}\left(k\frac{\partial T}{\partial z}\right)+Q$$

where $c_{p}$ is the constant-pressure specific heat capacity of argon; k is the thermal conductivity of argon; $F_{r}$ and $F_{z}$ are the volumetric forces in the *r*- and *z*-direction components, $F_{r}={\left(J\times B\right)}_{r}$, $F_{z}={\left(J\times B\right)}_{z}+\rho g$, and *J* are the current densities; *B* is the magnetic induction intensity; *g* is the gravitational acceleration; and *Q* is the source term of the energy equation.

In order to solve for the physical quantities of the electromagnetic field, it is necessary to introduce the current continuity equation as shown in Equation (5).

(5) $$\frac{\partial }{\partial z}\left(\sigma \frac{\partial \phi }{\partial z}\right)+\frac{1}{r}\frac{\partial }{\partial r}\left(\sigma r\frac{\partial \phi }{\partial r}\right)=0$$

Ohm’s law is shown in Equation (6).

(6) $$J_{r}=-\sigma \frac{\partial \phi }{\partial r},\;J_{z}=-\sigma \frac{\partial \phi }{\partial z}$$

Ampere’s circulation theorem (math.) is shown in Equation (7).

(7) $$B=\frac{\mu_{0}}{r}\int_{0}^{r}J_{z}rdr$$

where σ is the argon conductivity; ϕ is the electric potential; *r* and *z* are the radial and axial coordinates, respectively; $J_{r}$ and $J_{z}$ are the radial and axial components of the current density, respectively; *B* is the magnetic induction; and $\mu_{0}$ is the vacuum permeability.

### 2.3. Boundary Condition

**Figure 2:**
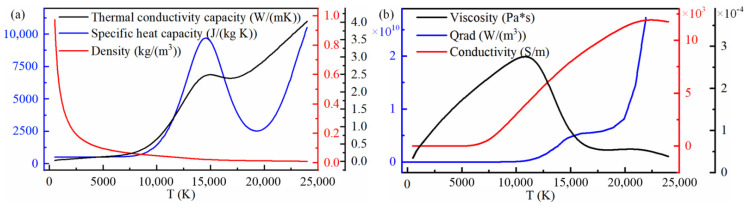
Thermal physical properties. (**a**) Relations between thermal conductivity capacity, specific heat capacity and density. (**b**) Relations between viscosity, qrad and conductivity.

The heat loss by arc radiation is denoted by $S_{R}=C_{0}T_{R}^{4}$ [25], where $C_{0}$ is the radiation coefficient; and $T_{R}$ is the temperature of the radiating body. The source term *Q* of the energy equation is shown in Equation (8).

(8) $$Q=\frac{J_{z}^{2}+J_{r}^{2}}{\sigma }+\frac{5k_{B}}{2e}\left(J_{z}\frac{\partial T}{\partial z}+J_{r}\frac{\partial T}{\partial r}\right)-S_{R}$$

where σ is the argon conductivity; *r* and *z* are radial and axial coordinates, respectively; $J_{r}$ and $J_{z}$ are the radial and axial components of the current density, respectively; $k_{B}$ is Boltzmann’s constant; *e* is the electron charge; and *T* is the thermodynamic temperature of argon. Thermal physical properties are shown in Figure 2.

## 3. Empirical Verification

**Figure 3:**
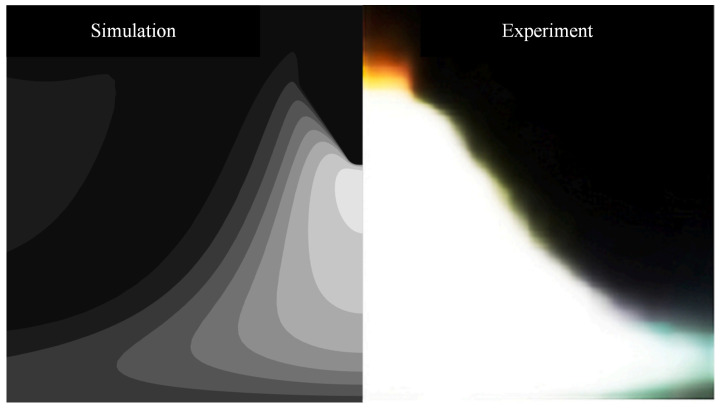
Experimental verification of arc shape.

Due to experimental constraints, this paper uses the experimental results of Liu et al. to validate the correctness of the proposed model. In the experimental verification process, the arc current was set to 100 A, consistent with the settings of Liu et al. [4]. The arc shapes obtained from numerical calculations and experiments are shown in Figure 3. As can be seen from Figure 3, the arc shape exhibits a bell-shaped profile. As shown in Figure 3, although there is some error between the arc morphology calculated by the proposed model and the experimental arc morphology, they generally agree well. The reason for the discrepancy between the model and experimental results is primarily due to differences between the thermal-physical properties used in numerical calculations and actual thermal-physical properties. Additionally, the numerical calculation process makes necessary assumptions about arc welding, which also differ from actual arc welding conditions. Therefore, the arc geometry calculated by the proposed model differs from the actual arc geometry.

## 4. Arc Numerical Calculation Results

### 4.1. Characterisation of the Effect of Current on Potential, Temperature and Voltage Fields

#### 4.1.1. Characterisation of the Effect of Current on the Distribution of the Potential Field

**Figure 4:**
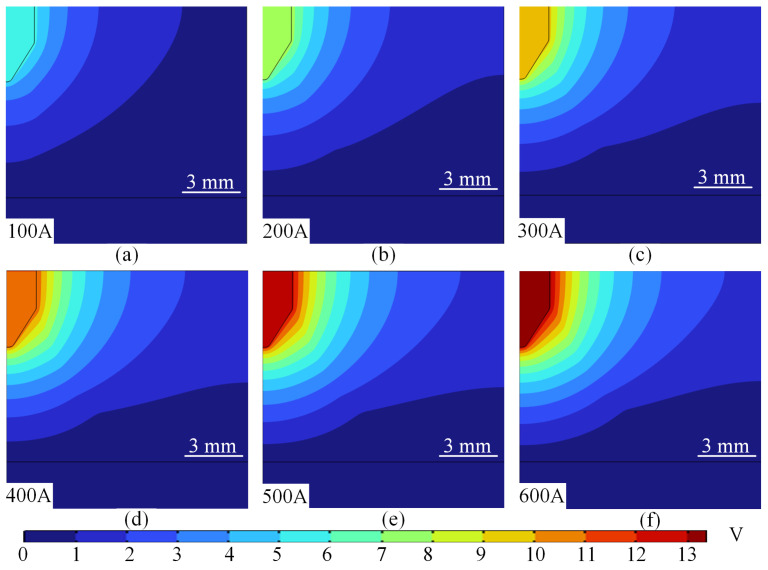
Arc potential field distribution at different values of current. (**a**) 100 A. (**b**) 200 A. (**c**) 300 A. (**d**) 400 A. (**e**) 500 A. (**f**) 600 A. As the current increased to 400 A, the potential gradient increased significantly, with the potential in the core area rising to 11 V and the rate of decrease in the periphery accelerating. When the high potential region extends to the boundary of the arc sheath layer, the contour lines are dense in the sheath layer region as shown in Figure 4d. This can be explained by the magnetic flux density, and the flux density mode distribution at different currents is shown in Figure 5. From Figure 5a–f, it can be seen that the magnetic flux density gradually extends towards the bottom of the tip of the wire. At this time, the Joule heat feedback effect is significant, and the increase in current density leads to a significant increase in Joule heat, and the conductivity rises with temperature, weakening the local potential gradient. At the same time, the plasma flow effect is enhanced, and the Lorentz force drives the argon flow to form a convective electric field, resulting in the potential spreading outward.

The flow rate of argon, the arc shielding gas, is constant at 30 L/min, and the potential changes under different currents, as shown in Figure 4. From Figure 4a–f, it can be seen that the potential at the tip of the wire increases with the increase in current, and the potential gradually decays to the surrounding protective gas with the tip of the wire as the centre. Meanwhile, when the current is 100 A, the potential distribution shows a gentle gradient characteristic, with a core potential of about 5.31 V and a peripheral potential that gradually decreases to near zero. In addition, the overall symmetry of the potential is good, the contours are distributed in concentric circles, and the high potential region is concentrated in the centre of the electrode as shown in Figure 4a. The reason for this change in the potential of the arc is mainly dominated by ohmic losses. When the current density is low, the Joule heating effect is limited, the conductivity change is not obvious, and the potential gradient is determined by the uniform current distribution.

**Figure 5:**
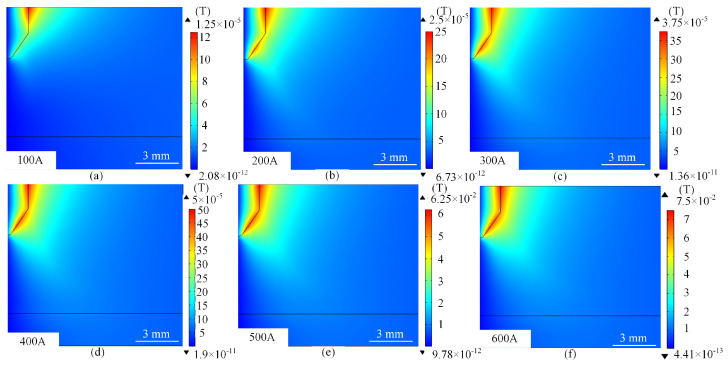
Mode distribution of magnetic flux density for different values of current. (**a**) 100 A. (**b**) 200 A. (**c**) 300 A. (**d**) 400 A. (**e**) 500 A. (**f**) 600 A. In order to observe more intuitively the change in potential with current, the characteristic curves of arc potential distribution under different current levels are given in Figure 6. The current potential is essentially the result of electromagnetic-thermal-fluid multi-physical field coupling: at low currents, the potential distribution is dominated by Ohm’s law, showing symmetric and uniform characteristics; at medium and high currents, the Joule thermal feedback, plasma flow and magnetic field distortion work together, resulting in a nearly linear enhancement of the potential as shown in Figure 6.

When the current reaches 500 A–600 A, the potential gradient becomes extremely steep, with the potential in the core area as high as 12.3–13.4 V. The potential at the edges drops rapidly to zero, the contour lines bifurcate or distort, and the high-potential area covers the periphery of the arc. At this time, the strong magnetic field distortion effect increases significantly with the current, and the magnetic flux density increases significantly. When the current increases from 100 A to 600 A, the range of magnetic flux density increases accordingly, and the peak value increases significantly. As shown in Figure 5. The peak magnetic flux density increases from 0.0000125 T to 0.075 T, as shown in Figure 5a,f, and gradually covers the tip of the wire. The superposition of the current self-consistent magnetic field with the applied magnetic field leads to the distortion of the magnetic lines of force and shifts the current path. The plasma turbulence is enhanced, and a tiny negative potential is formed in the edge region due to charge separation, while the high temperature leads to material loss on the electrode surface, and the local fluctuation of conductivity exacerbates the complexity of the potential distribution.

**Figure 6:**
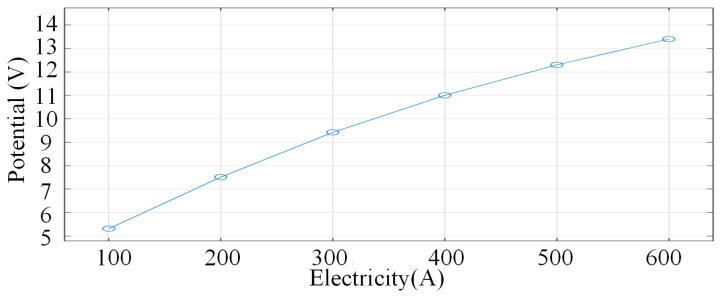
Characteristic curves of arc potential at different values of current.

During arc welding, the potential distribution is dominated by the coupling of current density *J* and conductivity *σ* showing significant electrode region differences: cathode region due to strong electric field driven hot electron emission, steep potential gradient, sheath effect compresses the current path, the Joule heat concentration leads to a sharp increase in the local temperature, and the increase in conductivity partially offsets the increase in the current density, forming a dynamic equilibrium; anode region is affected by the plasma flow and the arc constriction effect, and the potential distribution is overall flat, but the edges may be triggered by the concentration of the current path with a secondary. The overall potential distribution in the anode region is flat due to the plasma flow and the arc contraction effect, but the secondary potential peaks may be triggered at the edges due to the concentration of the current path, reflecting the synergistic compression effect of the geometrical constraints and the Lorentz force (*J × B*).

#### 4.1.2. Characterisation of the Effect of Current on the Temperature Field Distribution

**Figure 7:**
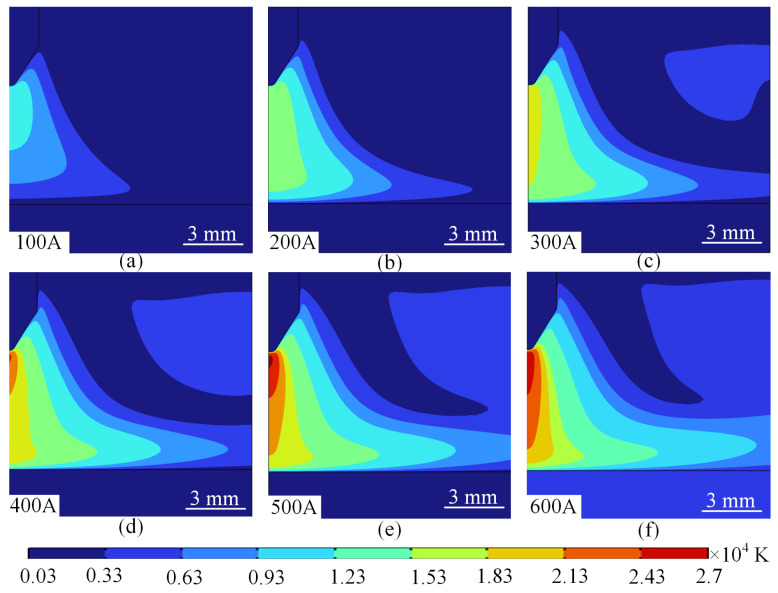
Arc isotherms at different values of current. (**a**) 100 A. (**b**) 200 A. (**c**) 300 A. (**d**) 400 A. (**e**) 500 A. (**f)** 600 A.

Figure 7 demonstrates the arc temperature field distribution characteristics at different currents. As a whole, the arc temperature increases with the increase in current and the temperature gradient gradually increases, as shown in Figure 7a–f. The reason for this change in the arc temperature can be explained by the arc energy; as the current increases, the energy consumed by the arc per unit of actual increases, leading to an increase in the temperature of the arc plasma. In addition, as the temperature increases, the resistivity of the arc argon increases and the efficiency of the conversion of electrical energy into thermal energy increases. Therefore, as the current increases, the temperature of the arc increases.

When the current is 100 A–200 A, the arc temperature gradient is flat, the maximum temperature of the plasma appears below the bottom of the tip of the wire, the core temperature of the arc is about 10,000–15,000 K, and the peripheral temperature gradually decreases to the ambient temperature (about 300 K), the symmetry is good, and the isotherms show an irregular elliptical distribution, and the high temperature zone concentrates on the tip of the electrode as shown in Figure 7a,b. At this time, the Joule heat is limited, the current density is low, the Joule heat generation rate is slow, the heat dominates the diffusion through heat conduction, the convective heat dissipation is weak, the plasma flow rate is low, the thermal conductivity of the material is stable, and the thermal conductivity of the copper electrode maintains the effective heat dissipation.

As the current increases to the middle 300 A–400 A, the temperature gradient is significantly enhanced, the core temperature rises to 182,00–22,000 K, the peripheral temperature drop rate slows down, the high temperature zone expands, the isotherm spreads outward, covering the arc sheath layer region isotherm appears bifurcated or twisted, there may be local hot spots, as shown in Figure 7c,d. At this time, the current density increases, the Joule heat rises significantly, the local heat accumulation intensifies, the high temperature leads to the elevation of argon conductivity, which further increases the Joule heat generation, and the Lorentz force drives the flow rate to increase, taking away the edge heat and forming a temperature gradient.

**Figure 8:**
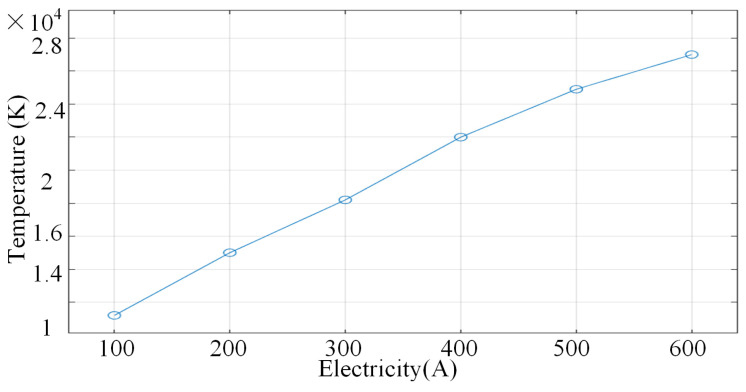
Temperature characteristic curves at different currents.

When the current intensity rises to 500 A–600 A, the isotherm bifurcates or twists, and the local hot spot area gradually increases. At this time, the Joule heat increases dramatically, the plasma turbulence enhances the heat mixing, the thermal conductivity decreases leading to local heat accumulation, and the core temperature of the arc tip reaches as high as 24,900–27,000 K. Figure 8 shows the temperature characteristic curves under different currents. The evolution mechanism of the temperature field at different current levels can be summarised as follows: at low currents, the temperature distribution is dominated by heat conduction, showing symmetric and gentle characteristics. At medium and high currents, the Joule heat and plasma flow together drive the temperature gradient to grow linearly, as shown in Figure 8. The temperature characteristic curves at different currents show that the stability and energy efficiency of the temperature field can be balanced by selecting high thermal conductivity electrodes, regulating the argon flow and optimising the magnetic field.

#### 4.1.3. Characterisation of the Effect of Current on the Pressure Field Distribution

**Figure 9:**
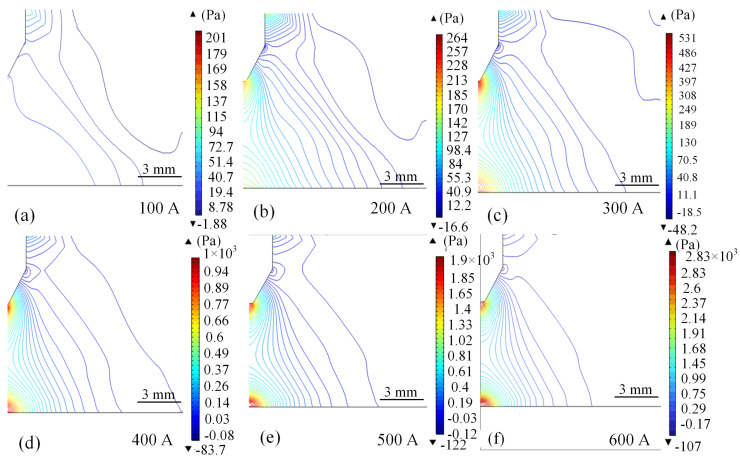
Arc pressure distribution at 100 A–600 A, respectively. (**a**) 100 A. (**b**) 200 A. (**c**) 300 A. (**d**) 400 A. (**e**) 500 A. (**f**) 600 A.

**Figure 10:**
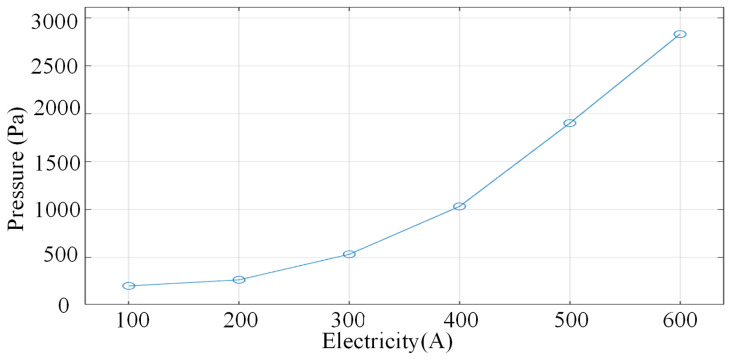
Arc pressure characteristic curves for different values of current.

Figure 9 and Figure 10 show the arc pressure cloud and the characteristic curve of arc current-pressure relationship under different current strengths, respectively. From Figure 9a–e, it can be seen that as the current increases the pressure on the arc tip and the substrate undergoes obvious changes, and the substrate directly below the wire circumference undergoes the greatest change. With the increase in arc current, the substrate high pressure area quickly to the wire axis extension line and the intersection of the substrate line close, and away from the centre of the pressure flow line gradually to the centre and the arc pressure maximum value is also increasing. In the current less than 200 A wire tip pressure zone increased significantly faster than the growth rate of the substrate high-pressure zone, the bottom of the wire tip pressure increased by nearly 56 Pa.

When the current is increased from 300 A to 500 A, there is not much difference between the pressure zone at the tip of the wire and the maximum pressure at the base plate. Through the analysis, it can be seen that when the current is increased from 100 A to 600 A, the intensity of the arc pressure field shows a nonlinear increase when the working current is 100 A, the maximum pressure value is about 200 Pa, as shown in Figure 9a. When the current is increased to 600 A, the maximum pressure value reaches 2830 Pa, as shown in Figure 9f, which is an increase of about 14 times, and the increase is gradually increasing. This phenomenon arises from the growth of the square relationship between the Joule thermal power and the Lorentz force density due to the increase in the current, as shown in Figure 10.

### 4.2. Characterisation of the Effect of Argon Flow Rate on the Physical Field Distribution

#### 4.2.1. Effect of Argon Flow Rate on Temperature Field Distribution

**Figure 11:**
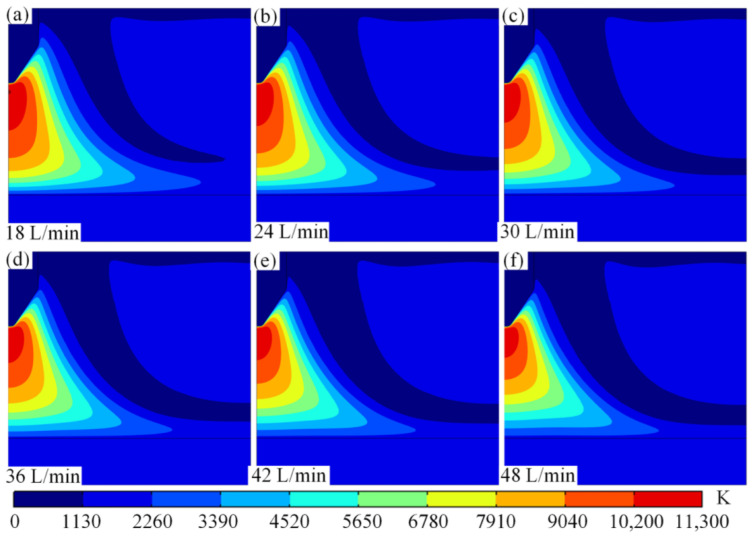
Arc temperature distribution for different flow rate values. (**a**) 18 L/min.xxx. (**b**) 24 L/min. (**c**) 30 L/min. (**d**) 36 L/min. (**e**) 42 L/min. (**f**) 48 L/min.

The arc temperature distribution at a current of 100 A and different argon flow rates is shown in Figure 11. When observing Figure 11, it is easy to see that the argon flow rate has a significant effect on the arc core temperature, the thermal boundary layer thickness and the electrode surface thermal management. When the flow rate is increased from 18 L/min to 24 L/min, the core temperature of the arc is maintained at about 11,300 K, and the high temperature zone is more concentrated, mainly in the area between the tip of the wire and the substrate, and the thickness of the thermal boundary layer is about 3.2 mm, and a local hot zone appears on the upper right side of the axis of the wire, with the temperature of about 4100 K as shown in Figure 11a,b. At this time, the phenomenon is because the argon inlet flow rate is low, the forced convection effect is relatively weak, the heat is mainly diffused to the periphery through the heat conduction mechanism, resulting in the electrode surface temperature as high as about 3000 K, the cooling efficiency is low.

As the flow rate increases to 30 L/min, the core temperature of the arc begins to decrease to about 11,200 K, as shown in Figure 11a. As the argon flow rate increases, the forced convection effect is significantly enhanced, pushing the low-temperature plasma to the anode, and the flow rate vector shows obvious flow consistency and a gradual decrease in the area of the local thermal region. The high-temperature region gradually extends inward, and the thickness of the thermal boundary layer is slightly reduced, as shown in Figure 11c. This phenomenon shows that a moderate increase in argon flow rate can effectively improve the thermal management performance of the electrode.

When the flow rate is further increased to a flow rate range of 42 L/min–48 L/min, the turbulent mixing effect is significantly enhanced, the core temperature of the arc is further reduced to about 11,100 K, and the thickness of the thermal boundary layer is compressed to about 1.2 mm. The temperature distribution gradually becomes homogeneous, and the diffusion range of the high-temperature region is significantly reduced. The surface temperature of the electrode is also reduced to about 1900 K, and the cooling efficiency is improved by about 36%, and the area of the local thermal region is almost unchanged, as shown in Figure 11d–f. That is to say, when the argon gas flow rate is higher, the turbulence effect not only enhances the mixing and diffusion of heat, but also significantly improves the cooling efficiency of the electrode. This phenomenon shows that the increase of argon flow rate has a significant modulating effect on the arc temperature field. For every 6 L/min increase in flow rate, the core temperature decreased by 5% to 7% on average. At the same time, the thickness of the thermal boundary layer decreases with the increase in flow rate, and the diffusion range of the high-temperature region is also reduced. For the electrode surface thermal management, the electrode surface temperature decreased from 3000 K to 1900 K when the flow rate increased from 18 L/min to 48 L/min, and the cooling efficiency was significantly improved. By optimising the argon flow rate, the distribution characteristics of the arc temperature field can be effectively controlled.

**Figure 12:**
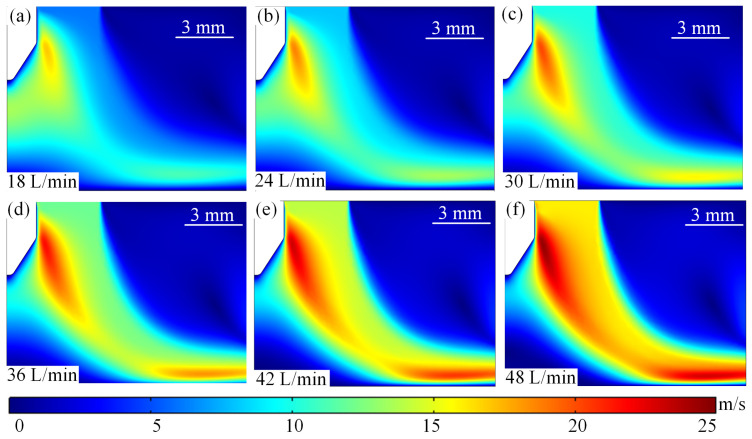
Distribution of arc fluid velocity field under different argon gas flow rates. (**a**) 18 L/min. (**b**) 24 L/min. (**c**) 30 L/min. (**d**) 36 L/min. (**e**) 42 L/min. (**f**) 48 L/min.

Figure 12 shows the arc fluid field distribution at different argon flow rates. From Figure 12a–f, it can be seen that the maximum flow velocity of the arc plasma increases gradually with the increase in the inlet argon flow rate. When the inlet argon flow rate is 18 L/min, the maximum flow velocity of the plasma is mainly distributed near the edge of the wire tip. At the same time, with the increase in the inlet argon flow rate of high-speed plasma began to shift to the right side of the wire axis direction, and the high-speed plasma region between the bottom of the wire tip and the substrate gradually decreases. When the inlet argon flow rate increases to 36 L/min, the maximum flow rate reaches 21.7 m/s, and the high-speed particle region is basically formed, as shown in Figure 12d. When the inlet argon flow rate increases to 48 L/min, the plasma flow rate will further increase and the high-speed particle region is gradually contracted, as shown in Figure 12f. Therefore, it can be introduced that the flow rate increases → forced convection heat dissipation is enhanced → the temperature of the arc region is reduced, and the cooling efficiency of the electrode surface is improved. Arc morphology can be controlled by high-velocity airflow, which can stretch or deflect the arc and change its contact point with the electrode. At high flow velocities, turbulence may be induced, enhancing mixing and heat transfer.

#### 4.2.2. Effect of Argon Flow Rate on Pressure Field Distribution

**Figure 13:**
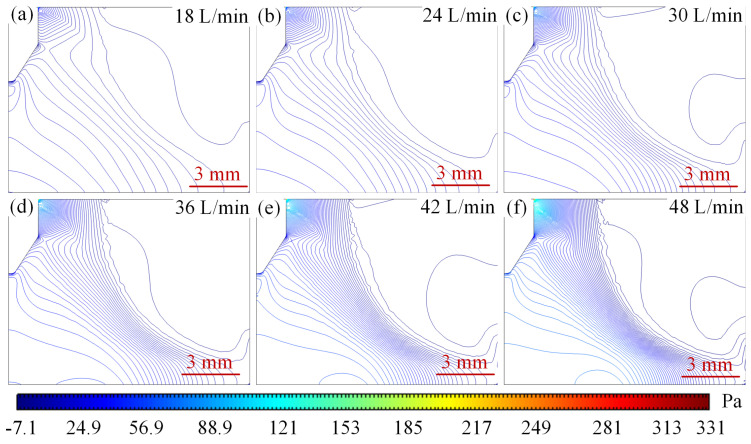
Arc pressure field distribution at different argon flow rates. (**a**) 18 L/min. (**b**) 24 L/min. (**c**) 30 L/min. (**d**) 36 L/min. (**e**) 42 L/min. (**f**) 48 L/min.

Figure 13 shows the arc pressure field distribution at different argon flow rates. When the inlet argon flow rate is 18 L/min, the maximum pressure is mainly distributed at the junction of the bottom of the wire tip and the argon inlet, with a peak pressure of 118 Pa as shown in Figure 13a. Other areas of pressure density distribution are looser. Argon gas expansion and density reduction triggered by the high temperature region of the arc, the formation of vertically upward natural convection heat plume, the pressure gradient is gentle, the core of the arc and the electrode near the pressure difference is small, showing a ‘low centre, high edge’ distribution characteristics; when the flow rate increased to 36 L/min, the location of the high-pressure region did not undergo a major change in the peak pressure with the entrance to the Argon flow rate increases to 247 Pa, the pressure density of significant changes in the pressure directly below the tip of the wire and the right side of the argon inlet pressure gradually to the middle of the convergence, the formation of an approximate diagonal distribution and the gradual increase in pressure density, as shown in Figure 13b–d. Inlet kinetic energy enhancement to promote the arc to the anode extension, the core temperature is reduced and the thermal boundary layer is compressed to 2 mm, the tip of the wire and the substrate to form a significant pressure difference, driving the plasma accelerated flow; with the entrance of the argon flow rate of 42 L/min, the tip of the wire negative pressure is also increased from the initial 3.38 Pa to 6.15 Pa, as shown in Figure 13a–e, to produce a new negative pressure region. Argon flow rate reaches 48 L/min when the pressure density in the middle further strengthens the negative pressure region to expand and converge to the middle, as shown in Figure 13f. After the flow enters the turbulent state, the vortex structure enhances the thermal diffusion, the core temperature of the arc further decreases, and the peak pressure on the anode surface reaches 331 Pa. The high-speed gas flow stretches the arc, resulting in an increase in its L/D ratio, and the morphology is elongated, and the dynamic instability is aggravated. The stepwise increase in the flow rate changes the energy transport mechanism, gradually shifting from natural convection to forced flow and turbulent mixing, and comprehensively regulating the thermodynamic and kinetic behaviour of the arc.

The reason for the above changes in the pressure field can be explained by the coupling of the Bernoulli effect, thermal expansion buoyancy and Lorentz force to regulate the arc pressure distribution: dominated by the Bernoulli effect, when the flow rate is increased from 18 L/min to 48 L/min, the kinetic pressure in the high flow rate region is increased so that the static pressure at the arc core is lowered from 7.1 Pa to 3.38 Pa, whereas the static pressure at the anode surface is significantly elevated by 331 Pa due to the kinetic energy-to-static pressure conversion; thermal argon expansion triggers buoyancy-driven natural convection at low flow velocity (18 L/min), forming a vertical thermal plume that leads to a significant low-pressure region below the arc, while the Lorentz force synergistically compresses the plasma with the high-speed flow at 48 L/min, intensifying the local high-pressure at the anode to 331 Pa. The flow state further affects the pressure distribution: the pressure gradient is smooth and symmetric in laminar flow at low flow velocity, and turbulence occurs at high flow velocity to enhance the dynamic pressure by vortex mixing at high flow velocity. Vortex mixing at high flow velocity enhances momentum exchange, resulting in high-frequency fluctuations in the pressure distribution, which comprehensively reflects the multi-physical field coupling characteristics of fluid kinetic energy, thermodynamic expansion and electromagnetic drive.

### 4.3. Comparison of Flow Velocity Sensitivity Under Different Currents and Its Guidance for Practical Applications

#### 4.3.1. Effect of Flow Rate Sensitivity on Temperature Field Distribution Characteristics at Different Current Intensities

**Figure 14:**
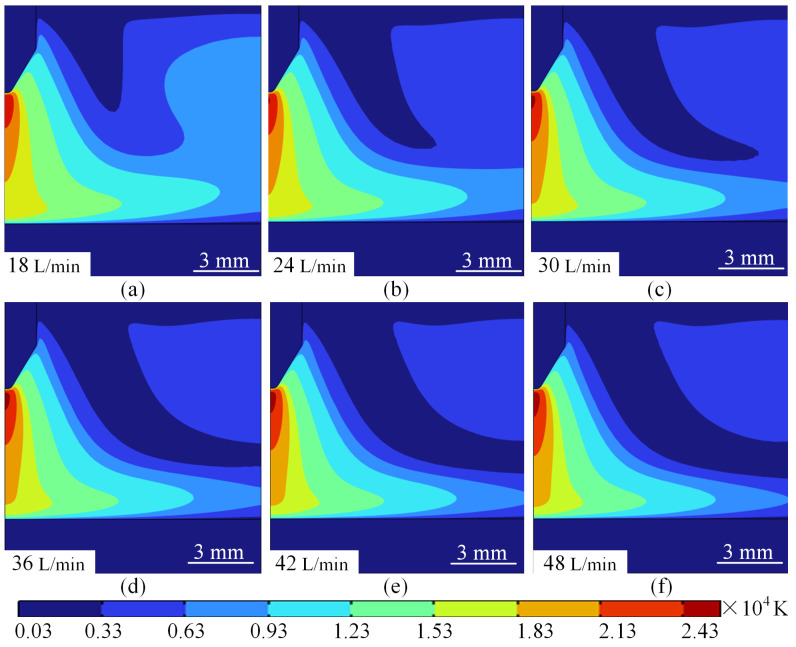
Arc isotherms at different argon flow rates at 500 A. (**a**) 18 L/min. (**b**) 24 L/min. (**c**) 30 L/min. (**d**) 36 L/min. (**e**) 42 L/min. (**f**) 48 L/min.

Under different current strengths, the same inlet flow rate to produce the arc temperature there are obviously different differences. Figure 14 for the 500 A current conditions of different inlet argon flow rate arc isotherm can be seen in the flow rate of the impact of the arc temperature due to different current strengths show significant differences: high current (500 A), with the increase in the flow rate of the high-temperature plasma area is gradually narrowed and to the tip of the wire area closer to the periphery of the plasma temperature is also gradually reduced at the same time, the rate of temperature reduction is also gradually accelerated, as shown in Figure 14a–f.

**Figure 15:**
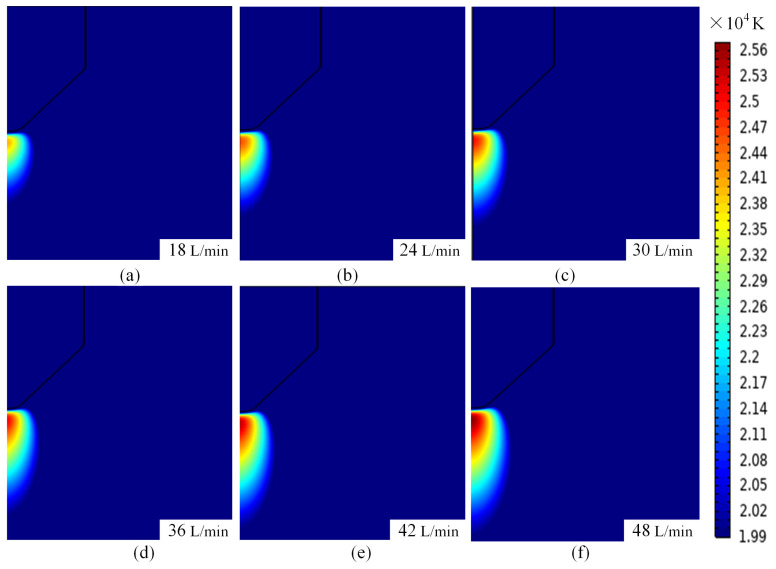
Arc tip temperature at different argon flow rates at 500 A. (**a**) 18 L/min. (**b**) 24 L/min. (**c**) 30 L/min. (**d**) 36 L/min. (**e**) 42 L/min. (**f**) 48 L/min.

It is worth noting that when the current is 500 A, as the argon gas flow rate increases, the temperature of the area directly below the welding wire tip and the substrate core abnormally rises from 24,300 K to 25,700 K, with the temperature gradient intensifying and the high-temperature region elongating, as shown in Figure 15a–f. This finding has not yet been discovered by other researchers. The change in arc temperature at a current of 500 A can be explained from two aspects. First, Joule heating: In the fractal arc heat formation mechanism and the mechanism of argon gas compressing the arc, the Joule heating power increases dramatically at high currents (Q ∝ I^2^), while the turbulent mixing effect enhances the thickness of the thermal boundary layer, weakening heat dissipation capacity and leading to temperature rise dominated by energy accumulation. This precisely reflects the nonlinear coupling characteristics between current and flow velocity. When the current is 100 A, the flow velocity increases from 18 L/min to 48 L/min, and the enhanced forced convection causes the arc core temperature to decrease from 11,300 K to 11,100 K, while the thermal boundary layer thickness compresses from 3.2 mm to 1.2 mm, resulting in a gradual improvement in heat dissipation efficiency, as shown in Figure 11. Second, the compression of the plasma by the protective gas: When the current is 500 A, the plasma density between the tungsten electrode and the substrate increases. Under the pressure of the protective gas argon, part of the plasma moves closer to the central axis, while another part moves away from it. This results in a more pronounced separation of plasma toward and away from the central axis as the protective gas flow rate increases. Consequently, the plasma closer to the central axis becomes more concentrated, and this concentration inevitably leads to an increase in arc temperature at the central axis of the arc. This is why the temperature rises as the protective gas flow rate increases at a current of 500 A.

**Figure 16:**
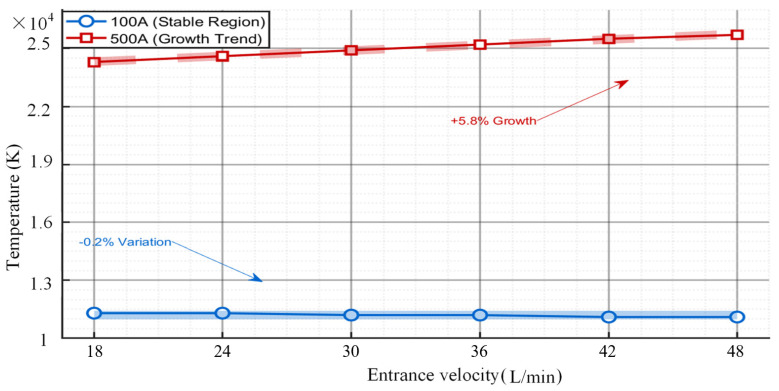
Variation curve of temperature with argon flow rate under different currents.

Figure 16 shows the temperature versus airflow velocity at different currents. As can be seen from Figure 16, the effect of airflow velocity on the temperature field varies considerably at different currents. At an operating current of 100 A, there is only a small fluctuation (−0.2%) in the temperature as the velocity increases, which indicates that the system has good heat transfer performance and low sensitivity to flow velocity, which makes it suitable for applications where constant temperature control is required. However, when the current is 500 A, the internal temperature rise increases sharply with the increase in flow rate (+5.8% increase), a phenomenon suggests that a reduction in the efficiency of heat dissipation can cause a localised region of temperature rise or a sudden change in the temperature gradient, requiring a dynamic flow control or enhanced heat dissipation to prevent this. This difference suggests that the current strength is achieved by regulating the heat production balance, which directly affects the temperature sensitivity of the flow rate and the stability of the temperature field.

**Figure 17:**
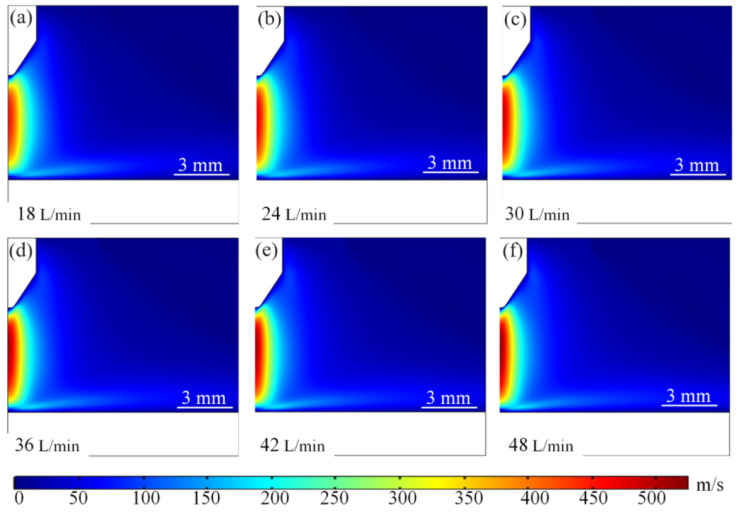
Fluid velocity plots at different flow numbers for 500 A. (**a**) 18 L/min. (**b**) 24 L/min. (**c**) 30 L/min. (**d**) 36 L/min. (**e**) 42 L/min. (**f**) 48 L/min. At low current (100 A), the flow rate increase tends to saturate the temperature suppression effect from 11,300 to 11,100 K, and the heat dissipation efficiency is limited by the low Joule heat base, which is applicable to the low-energy steady state scenario; under the high-current situation when the current reaches 500 A, the flow rate increases through the shrinkage of the arc, and the radius of the arc shrinks from 3.2 to 1.2 mm. The turbulence kinetic energy accumulates with the anode high-speed impingement effect triggering a drastic increase in the Joule heat turbulent kinetic energy build-up and high-speed anode impact effect lead to a dramatic increase in the Joule heat density, resulting in a paradoxical increase in core temperature from 24,300 K to 25,700 K, exposing the essential contradiction between energy density and heat dissipation capability. This phenomenon reveals the complexity of multi-physical field coupling in extreme operating conditions: the strong nonlinear competition between Lorentz force, fluid kinetic energy and heat transfer may trigger thermal runaway, and it is necessary to optimise and balance the energy transport paths through multi-scale synergy in order to inhibit local overheating.

The current is constant at 500 A, and the flow rate of plasma under different argon flow rates is shown in Figure 17. From Figure 17a–f, it can be seen that although the flow rate of the inlet argon is gradually increasing, the high-speed region of the plasma still accumulates at the tip of the wire. The Lorentz force dominates the arc contraction, and the arc diameter is compressed from 3.2 mm to about 1.2 mm, the energy density increases dramatically, and the Joule heat power density increases dramatically, which counteracts the heat dissipation effect of the flow rate increase. At the same time, the kinetic-thermal energy conversion between the energy accumulation in the contraction area and the high-speed plasma impacting the anode further aggravates the core temperature from 24,300 K to 25,700 K, forming the anomalous phenomenon of ‘flow rate increase, temperature rise’, highlighting the strong nonlinear competition between electromagnetic force and hydrodynamic force under high current.

#### 4.3.2. Flow Velocity Sensitivity to Pressure Field Distribution Characteristics at Different Current Intensities

**Figure 18:**
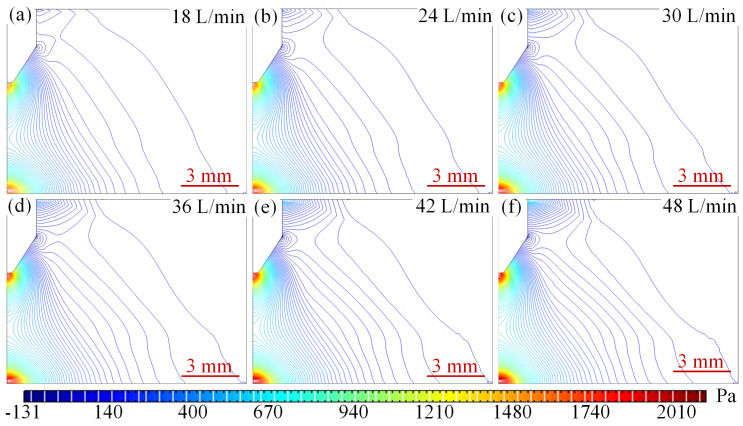
Pressure distribution under different argon currents at 500 A. (**a**) 18 L/min. (**b**) 24 L/min. (**c**) 30 L/min. (**d**) 36 L/min. (**e**) 42 L/min. (**f**) 48 L/min.

The pressure distribution of the plasma at different argon flow rates with a constant current of 500 A is shown in Figure 18. From Figure 18a–f, it can be seen that the peak pressure increases as the current increases, and the negative pressure also increases gradually. At a current of 100 A, the flow rate increases from 18 L/min to 48 L/min, the peak anode pressure rises from 118 Pa to 331 Pa, and the total pressure changes 213 Pa, with an average gradient of 21.3 Pa/(m/s), and the pressure gradient is gentle and the peak is concentrated in the junction area between the argon inlet and the bottom of the wire tip (Figure 18a), indicating that the flow kinetic energy plays a dominant role in the inlet local kinetic pressure enhancement.

**Figure 19:**
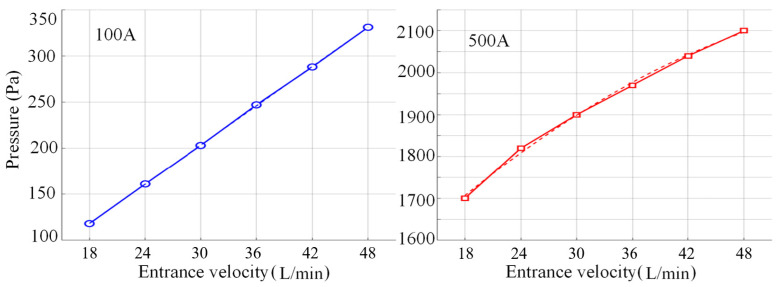
Variation curve of pressure with argon flow rate under different currents.

When the working current is 500 A, the peak anode pressure in the same flow rate range surges from 1700 Pa to 2100 Pa, with a total pressure change of 400 Pa and an average gradient of 40 Pa/(m/s), as shown in Figure 19. The pressure density is significantly enhanced, and the peak is concentrated between the wire tip and the substrate, as shown in Figure 18a–f. The mechanism originates from the strong compression effect of the Lorentz force on the arc under high current and the synergistic effect of turbulent kinetic energy, which leads to a dramatic increase in the Joule thermal power density, and further drives the plasma kinetic energy to the electrostatic pressure conversion efficiency to improve the formation of a high gradient, high concentration of the pressure distribution characteristics, which reveals the amplification of the current intensity on the electromagnetic-fluid coupling effect.

Under the conditions of small current (100 A) and large current (500 A), the energy distribution of the arc and the mechanism of pressure formation are very different: under the conditions of small current, the energy density of the arc is very small, evanescent, with a large conical structure; the energy is dispersed, and the high-voltage core area cannot be formed. Under low-speed airflow conditions, the sudden change in the inlet structure produces a local vortex or collision, which converts the kinetic energy in the inlet into pressure energy, resulting in a peak of up to 118–331 Pa at the arc tip due to the lack of energy; under high current, due to the increase in current, the plasma inside the plasma is compressed into the cone, which produces a high-density plasma, and the Joule heat power density increases sharply; under the combined effect of the Bernoulli effect and turbulence, the kinetic energy is efficiently converted into the tip-pressure energy, which reaches 2100 Pa; meanwhile, the amplification effect of the current field on the electromagnetic field-fluid field-geometry multi-field coupling is highlighted on this basis.

At a small current of 100 A, the energy of the arc is dispersed and covers a large area, and the local pressure concentration in the inlet region due to the airflow impact and vortex effect results in insufficient energy accumulation at the tip of the arc. When the current reaches 500 A, the Joule thermal power density (*Q* ∝ *I*^2^/A) excited by the Lorentz force increases dramatically, which, together with the Bernoulli effect caused by geometrical contraction and the increase in turbulent energy, causes the energy and pressure in the flow field to accumulate at the tip. Under high current conditions, the multi-field coupling effects of Joule heat, electromagnetic compression and turbulence will greatly enhance the sensitivity of the air pressure to the flow field, thus revealing the complex nonlinear characteristics of the electric-electrical-fluid-thermal multi-field coupling in the extreme operating environment.

#### 4.3.3. Flow Velocity Sensitivity to Potential Field Distribution Properties at Different Current Intensities

**Figure 20:**
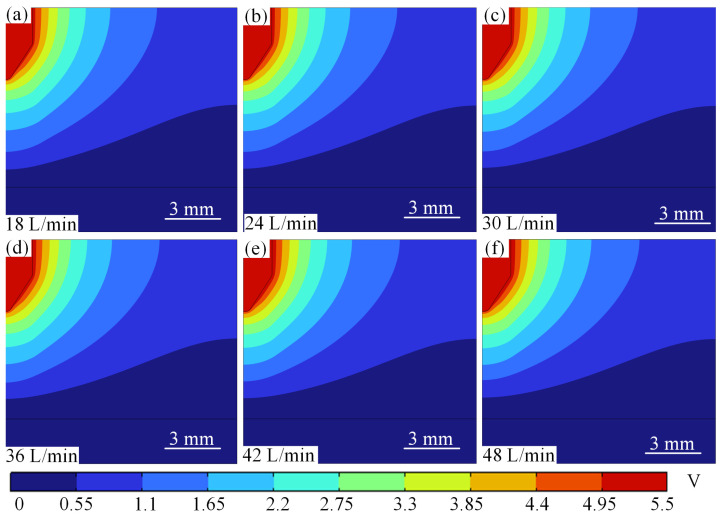
Potential distribution at different argon flow rates for 100 A. (**a**) 18 L/min. (**b**) 24 L/min. (**c**) 30 L/min. (**d**) 36 L/min. (**e**) 42 L/min. (**f**) 48 L/min.

When the current is constant at 100 A and the gas flow rate increases from 18 L/min to 48 L/min, the potential changes as shown in Figure 20. From Figure 20, it can be seen that the peak arc potential increases from 5.22 V to 5.41 V as the argon gas flow rate increases, with a gentle increase, as shown in Figure 20a–f. At low current (100 A), the increase in flow rate (18 → 48 L/min) reduces the temperature by forced convection (11,300 → 11,100 K), the conductivity decreases slightly, and the potential rises slowly only from 5.22 V to 5.41 V.

**Figure 21:**
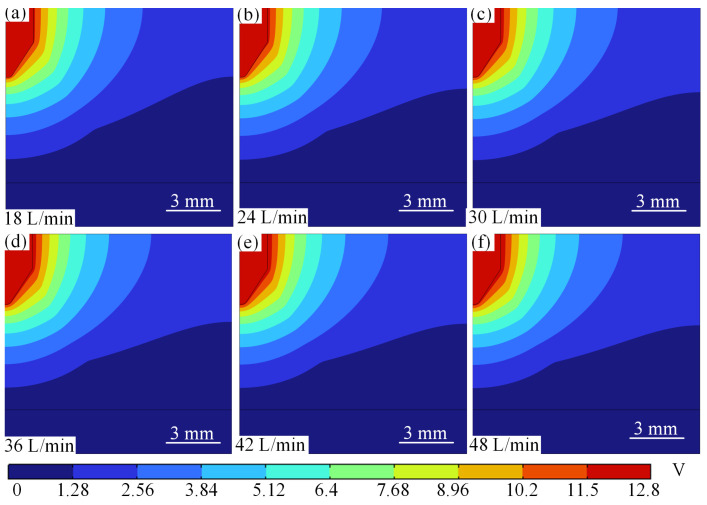
Potential distribution at different argon flow rates for 500 A. (**a**) 18 L/min. (**b**) 24 L/min. (**c**) 30 L/min. (**d**) 36 L/min. (**e**) 42 L/min. (**f**) 48 L/min.

The variation in voltage with argon flow rate at a current of 500 A is shown in Figure 21. At high current (500 A), the conductivity increases significantly with temperature, while the Joule heating power increases dramatically, resulting in a significant decrease in arc resistance due to the increase in conductivity and the reduction in arc cross-sectional area A, which counteracts the linear driving effect of the current on voltage (*V* = *IR*); the Lorentz force enhancement further compresses the arc to a smaller area, and the current density (*J* = *I/A*) surges, and the path centralisation further reduces the resistance and suppresses the voltage increase. At high currents, the increased flow rate exacerbates the arc contraction and Joule heat accumulation (24,300→25,700 K), and the increased conductivity dominates the resistance drop, resulting in a limited potential increase and only a voltage increase from 11.8 to 12.8 V.

**Figure 22:**
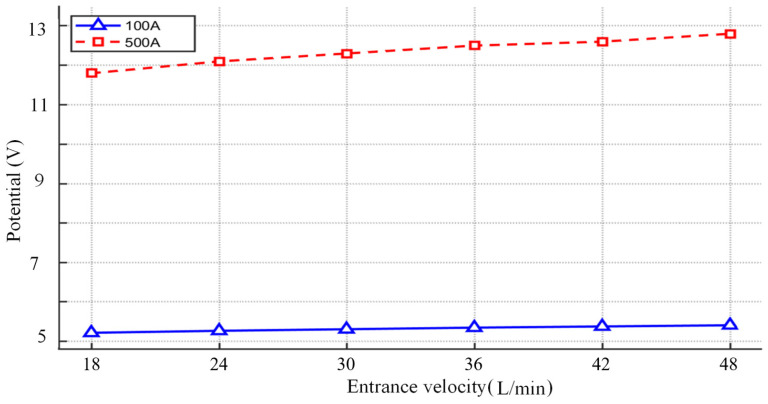
Curve of potential variation with argon flow rate at different currents.

The effect of flow rate on the peak arc potential shows a differentiated response at different current strengths: at low current (100 A), the flow rate increases from 18 L/min to 48 L/min, and the peak arc potential rises from 5.22 V to 5.41 V. The mechanism is that the increase in flow rate slightly reduces the arc temperature through forced convection, and the conductivity decreases slightly, resulting in a slow increase in potential, but the thermal-electrical coupling effect is weak due to the low Joule heat base. The potential increases from 11.8 V to 12.8 V in the same flow rate range at high current (500 A), and the potential gradient is slightly higher than that of the low-current case, but the increase is still suppressed, as shown in Figure 22. The reason for this is that under high current, the conductivity due to the temperature increased from 24,300 to 25,700K, so that the effect of temperature on the decrease in resistivity, partially offset the current density due to the arc contraction caused by the increase in resistance, the formation of ‘flow rate increase→temperature rise→conductivity rise→resistance drop’ negative feedback equilibrium, weakening the trend of the linear growth of the potential. This phenomenon highlights the role of current intensity in regulating the electric–thermal–fluid coupling mechanism, which provides a key constraint for the voltage stability design of electric arc equipment.

#### 4.3.4. Guidance on Numerical Calculations for Practical Applications

Based on the above calculation results, it can be seen that at low current (100 A), the higher the shielding gas flow rate, the lower the arc temperature, and the high-temperature zone of the arc contracts toward the centre axis of the arc. At high current (500 A), as the shielding gas flow rate increases, the high-temperature zone of the arc compresses, and the arc temperature rises. At the same time, the temperature at the arc welding substrate and the arc pressure both increase with the increase in current. This also indicates that in practical applications, the arc temperature and arc size can be controlled by simultaneously increasing or decreasing the arc current and shielding gas flow rate. Additionally, the magnitude of the arc’s force on the molten pool can be adjusted by controlling the current magnitude and shielding gas flow rate. Furthermore, the numerical model constructed in this paper can be used to elucidate the sensitivity of various process parameters to temperature fields, pressure fields, and voltage magnitudes.

## 5. Conclusions

In this study, based on a finite element numerical model with multi-physics field coupling, we systematically analysed the regulation laws of current intensity and argon flow rate on the temperature field, pressure field, and potential field of the DC arc, revealing the key mechanisms of the arc behaviour. The main conclusions are as follows:

(1) When the current increases from 100 A to 600 A, the Joule thermal power grows with the square of the current (*Q* ∝ *I*^2^), driving the core temperature of the arc to rise sharply. The pressure field distribution exhibits spatial migration with current—the pressure peak is located in the argon inlet region at low currents, whereas at high currents, the pressure peak shifts towards the conical tip of the arc, and the gradient increases significantly due to the enhancement of the Lorentz force. In the potential field response, despite the large increase in current, the potential increase is relatively flat, which is due to the exponential increase in plasma conductivity caused by the high temperature, which effectively offsets the resistive effect triggered by the increase in current, forming a dynamic equilibrium.

(2) When the current is 100 A, the argon flow rate (18 L/min–48 L/min) has a weak inhibitory effect on the arc temperature, and the pressure field change is mainly dominated by the flow kinetic energy. At a current of 600 A, the increase in flow rate exacerbates the magnetic hoop contraction effect, leading to further arc contraction and energy accumulation at the tip, which triggers a local pressure surge and anomalous temperature increase.

(3) Due to the coupling effect of electrode geometry and arc morphology, the conical structure induces the acceleration of airflow and arc contraction. The tip becomes the core area of energy and pressure at high current, and the sudden change in inlet geometry at low current triggers local vortex formation and pressure concentration.

## Data Availability

The original contributions presented in this study are included in the article. Further inquiries can be directed to the corresponding author.
